# The Pulp and Treatment of Pulp Canals

**Published:** 1889-04

**Authors:** James Truman


### THE PULP AND TREATMENT OF PULP CANALS.[1]

BY JAMES TRUMAN, D.D.S.

The pulp of each tooth must be considered, not as a separate entity, but as a part of a living body essential to its life and a factor in its nourishment. While this is understood there seems to be more or less confusion arising from the want of due consideration being given to this fact. Its connections are so intimate with all the various tissues composing the tooth body, that a lesion affecting one may be regarded as having an influence more or less potent on all. The inflammatory results of irritation are divided under a number of distinct heads; yet a description of one will necessarily include them all, for they are intimately involved in one common origin. This inter-dependence one upon the other renders it all

[1] Read at the Tenth Anniversary meeting of the Odontological Society of Pennsylvania, Dec. 13, 1888.

the more necessary to have the subject clearly understood in its elementary and simple relations before proceeding to the consideration of the more complex.

The pathological state of the pulp, beginning with the first degree of irritation in thermal influence, to the closing scene when devitalization has destroyed the function of this original formative tissue, is one of interest to the practitioner and the successful management of which necessarily, marks the progressive stages of the dental profession. The reason for a word now upon it must be found in the fact that for a long period the treatment of the pulp has been relegated to the finished work of the profession, with the natural result, that practices have been developed wholly at variance with accepted views of a quarter of a century ago and not in accord with observed facts of more recent development. The ideas regarding the philosophy of treatment of pathological conditions have undergone a marked change in the past few years ; so great, indeed, that no apology need be offered for opening up the subject for further discussion. The day has certainly passed when the creosote and carbolic acid of a bygone time can longer assume to dominate the dental pharmaceutical preparations ; or that other remarkable period when empiricism took the place of intelligent reasoning. While it is true that it has passed away with many, it has not with all, and it is still a question whether there is any other branch of our speciality where more confusion abounds in regard to practice than exists here. While anything I may write may not clear up this subject it is to be hoped that there may be some light thrown upon it, and a few of the difficulties that have environed it removed by the discussion that ought to follow a subject of such vital practical importance.

Pulps have been the theme of endless comment, and their preservation and destruction have involved the expenditure of a vast deal of energy in the attempt to affect both in a satisfactory manner. From the time of Spooner, with his introduction of arsenic, to the days when Harris vigorously opposed and then subsequently conceded its value, to the period when it became the universal devitalizer, or to that later time when it was again denounced as unfit to use, and we were called to retain the life of the pulp at all hazards. When capping came in under new and superior modes, than were possible originally, to the period in which we now live, when the profession demands to know the reason for all these changes and asks for some explanation of the great variation in opinion on all topics in connection with the pulp and the treatment of canals.

The confusion of ideas must strike the casual observer as not altogether creditable to a so-called "learned profession." There must be a reason for this contrariety of opinion, based as it doubtless is on observation; yet we are no nearer a consensus of opinion to-day than we were forty years ago, and it is questionable whether we are as near the centre of truth on this subject as Maynard, Townsend, Westcott and others were, when they so beautifully illustrated their skill as manipulators in filling canals. I think the most candid observer must concede, as he reviews the work of that period, that the practice of canal treatment, as a whole, is inferior to that time. In examining this subject I have tried to divest myself of the feeling "that the former days were better than these;" but it is impossible to resist the conclusion, that while there has been great advances in other directions, there has been a retrograde in this. Indeed, we are where capping left us in the earlier days of 1850, with this improvement, that we know more of general pathological treatment. I am not, however, to discuss in this paper capping or its failures; but I wish to say this, that in exceptional and selected cases of extra vitality capping may be, and I know is, often a success; but in all cases where the pulp has assumed pathological conditions the retention of life must be of very rare occurrence if it ever takes place. This, if it be true, narrows the border of life in the pulp to such limits that we are forced to consider other modes and rely on pulp extirpation, until something better is developed out of the present chaos of professional thought worthy of our acceptance. I, therefore, leave the question of preservation entirely in the hands of others and confine myself exclusively to devitalization and the measures required for the subsequent preservation of the tooth for future usefulness.

The well-known modes of pulp destruction are: 1st. Forceful and immediate extirpation. 2d. The destruction by an escharotic, and 3d. By the slow process of death by inflammation. The first two have their advocates, and since the introduction of crowns, the number of operators who prefer driving out the pulp by wood and a sudden blow, forcing the pulp from its connections, are rapidly increasing. This heroic method is seriously advocated as a painless operation. That at times it does not seem to produce much pain must be acknowledged; but I have failed to find any one who could give a reason for this, and in the few cases where I have seen it tried, there was but one where there seemed to be no pain. The character of the tissue thus forced must lead us to look with suspicion on the assertion that no pain is felt. Statistics are wanting in regard to this, both as to the forceful removal, with or without the injection of a paralyzing agent, and until we have them it may be well to let the matter rest here with the usual conclusion, of all these controverted questions, that much may be said for and against the practice. Death by escharotics has not made much progress since the introduction of arsenic. No agent has been found to take its place, notwithstanding the efforts of the Germans, in their investigations, to substitue iodoform. In the use of arsenic, however, there has been an advance by the application of cocaine and iodoform to inflamed pulps. The former introduced by Dr. Kirk and the latter by myself. As a note of progress I may state that the use of iodoform, in this connection, has received confirmatory assurance of success from so many parts of the world, that the original conclusion arrived at may be regarded as fixed, and that we have at last a mode for the destruction of the pulp without pain, let the conditions be what they may.

Devitalization brings us directly into a maze of pathological difficulties, that it is not remarkable that the most courageous operator stands appalled at the possible consequences. We are met at once with a demand for our highest intelligence, for the greatest patience in treatment, with oftentimes discouraging results that baffle our best efforts for weeks and even months.

The relations of the pulp to the surrounding tissues are well understood, and it would be superfluous in me to enlarge upon them; but for a clear understanding of the argument I must say that the pulp by its prolongations maintains intimate relations with the periphery of the dentine, and by minute anastomoses with every part of this tissue, and, possibly, in some way as yet not clearly made out, with enamel and cementum. Its intimate connection with the pericementum needs only to be stated.

The death of the pulp is produced by, first, irritation. This necessarily follows any exposure. The causes of this incipient irritation are not far to seek. They exist in changes of temperature, of foreign matter collecting in masses—from food and broken down epithelial tissue—of the ever present low forms of life that become pathogenic upon the first favorable opportunity. These, and more, cause the exposed pulp to enter the second stage of inflammation in which the emigration of leucocytes and products of inflammation produce, with the congestion of the vessels, a hyperæmic condition that results in pressure on the sensory nerves, and final strangulation of all the sources of nutrition, and death follows. We now begin to understand how impossible it is that capping can be made a success except in absolutely normal pulps, and that these latter are exceedingly rare presentations. That it is possible to restore the pulp by starting the nutrient currents into normal·activity must be conceded; but that, as before stated, cannot be the general rule, the exceptions being found in cases of extraordinary resisting vital powers.

The destruction of the pulp is progressive as a rule; that is it begins at the surface and may remain for indefinite periods as a superficial inflammation; or, as I choose to call it, *superficial chronic pulpitis.* Devitalization proceeds slowly until finally the entire pulp is involved in destruction. From my observation I am inclined to think that it is rare to meet with the destructive inflammation in this organ so common in other tissues. Pus is to be found in pulps, and Black describes a condition he terms abscess of the pulp; but it would seem that the pus present must originate solely by emigration and not by the retrograde metamorphoses of the tissue. The death of the pulp from whatever cause, opens up to the operator the whole question of treatment, which I will endeavor to consider. I propose to examine the result from my point of view, following the various modes adopted and the possibilities of a successful termination.

By the forceful method the pulp is severed from its connections by a violent process. It is torn from the prolongations entering the tubuli and equally so from the sources of nutrition at the foramen. The canal is, to a large degree, freed from organic material. Is this true of the tubes? We have no evidence that the removal of the organic portion—the inner tubular tissue—has been disturbed at all. Its sources of nutrition have been cut off, for there are no facts to warrant the opinion that dentine retains its vitality after the pulp has been destroyed. Now it is in just such cases that the advocates of immediate filling hope for the best results, and it is here they should have it if it is ever possible. I am not prepared to deny the conclusions of those who regard immediate filling as the best. They have excellent reasons for its advocacy in this forceful removal. It is true that the organic tissue left in the canaliculi will gradually disintegrate, and slowly carbonize and eventually discolor the tooth; but if the canal be thoroughly filled I fail to understand how subsequent ill results, in other directions, can follow. This presupposes, of course, that antiseptic treatment has followed the removal of the pulp and continued until the canal filling is inserted. The favorable conditions found here do not exist under other modes of extirpation of this organ and, in the nature of the case, all immediate canal filling after the use of arsenic or death by inflammatory processes, is bad practice. This may seem a dogmatic assertion, and it *is* a positive statement; but in my judgment it is one easily proven from accepted facts.

Arsenic acts by paralyzing the nervous supply of the pulp, and thus in cutting off the sources of nutrition death follows. Decomposition, while more slowly produced, is sure to result from the application of this agent; besides it is important that immediately upon the removal of the visible pulp there should be applications to limit the action of any remaining arsenic absorbed. The immediate filling of the canal makes this impossible. Carbolic acid acts remarkably after the application of this agent. Clinically it has been noticed that it reduces the hyperæmic condition produced by arsenic, probably, by holding the agent in solution and preventing its further direct action. Time is, therefore, an important matter in the treatment of a canal after devitalization by this agent and immediate filling is inadmissible.

The third condition, that of death by the gradual process following either superficial or acute pulpitis, is one that has in all periods been regarded as the most difficult in the sense that the prognosis was always necessarily unfavorable. It may be well to consider the causes that lead up to this in cases of this kind. From the very beginning of irritation followed by a hyperæmic condition of the vessels of the pulp, there is an accumulation of micro-organic life. These low forms have been known and recognized for many years, indeed as far back as Leuwenhœck, who first described bacterial forms; but, as you all know, it has only been of recent years that they have been appreciated in a pathological sense, and this is due to the work of Schrœder, Pasteur, Bastian, Lister, Beale, Leber, Rottenstein, Koch, Miller and many others. There can be no inflammation of the pulp, or, for that matter, any inflammation of the mouth, that these forms do not take part as factors in the increase of the destructive effects. They are the invisible irritants both by their immediate presence and by their products.

This brings me to the consideration of the antiseptic treatment of canals. It has been but a comparatively limited period since the operator upon devitalized teeth regarded himself extremely fortunate if he succeeded in getting these teeth prepared for filling without an attack of pericementitis, and the danger was always imminent of an abscess with all its accompanying unpleasant conditions. The past decade has witnessed an entire change in this respect, and I wish to occupy your time for a brief period in a review of a portion of the work that has led up to this. It is well known that for an indefinite period little was understood in relation to this subject. The facts were apparent and clinically fairly comprehended; but no attempts were made to investigate the cause. The germ theory of disease began to claim attention; but, as you are well aware, it remained for years a theory, and not until the investigations alluded to were made, were we prepared to understand the true relations of a pulp to the surrounding tissues or the origin of putrefaction, the source of all the ills we knew so much about clinically and so little therapeutically. It would be impossible to state the number of years that first creosote and then carbolic acid held empiric sway over the professional mind. These two were the essentials in every dental medicine case and, with the tincture of iodine, held undisputed possession of dental offices until a very recent date. That the true action of these agents was but imperfectly understood is apparent from the fact that they thus held control for so long a period. The excuse for this state of things does not, however, longer exist, and no dentist is properly prepared to treat pathological conditions without a thorough comprehension of the whole subject, including the materia medica upon which so much now depends.

It has been said that while the origin of zymotic diseases has been more thoroughly investigated in the period named than in the prior centuries, yet the treatment has not advanced in equal degree. While this may be true in general practice, and while Koch may be able to demonstrate the bacillus of consumption and cholera, he nor his followers have succeeded in destroying these germs and preventing their ravages by any satisfactory agent. Prophylaxis is far in advance in this respect, and possibly the near future will demonstrate beyond a question that the future province of the physician and dentist will lie in preventing rather than in curing disease. The field is a wide and interesting one; but time will not permit elaboration on this point.

There was but little modification in the views of operators until the chemical changes occurring in putrescent pulps were examined into. Dr. Litch was the first, as far as I am aware, to call attention to these, and his intelligent description of the chemical relations of the decomposed pulp has remained as the basis of our knowledge on this question and a satisfactory solution of a hitherto difficult problem. This, coupled with the work of others, notably that of Miller and Black, has led up to a complete change in treatment among the progressive, intelligent minds of the profession. The fact that micro-organisms are a principal factor in putrefaction, indeed, that that process cannot go on without them, simplifies treatment with the dentist. More favored than the general practitioner, he can adopt the Listerine method at once, and prevent the development of germs in the early stages. It needs no argument here at this late day to prove this fact. If we were not in possession of the work of the distinguished gentlemen alluded to, we would still have our clinical experience to fall back upon as conclusive in the premises. It is safe to assert with entire positiveness, that a careful destruction of low forms of life in a canal, and subsequent sterilization of any instrument used, and then final exclusion, by proper agents, of all germs, that inflammation of the pericementum is an impossibility, and with this judicious treatment alveolar abscess would soon be placed amongst the lost diseases in properly treated patients. If, then, micro-organisms are essential to putrefaction, and the products of this chemical change produces pathological conditions in near and remote tissues, it is clear that the only canal capable of being filled at once after removal of the pulp is the one destroyed by forceful measures, and the earlier it is filled the better. All other cases require treatment.

The antiseptic agents are now so numerous, and are increasing so rapidly, that I would simply weary you with a recital of all of them, and will, therefore, confine myself to a few. It must be borne in mind that a true germicide is not always the most satisfactory agent to use, at least not in my opinion. An agent that can be used *ad libitum*, though possessed of but low germicidal powers, is superior, at times, to such a one as mercuric chloride. Again, one should be selected which combines, with its inhibiting power, the property of retaining its place without change for the longest possible time. This is, doubtless, the great value of iodoform in canals, that it cannot be broken up, but will retain its positive action for months and, possibly, for an indefinite period. Quinia, while weak in antiseptic qualities, is also of great value clinically, from its power of inhibiting pathogenic forms. The number of antiseptics has become so numerous, and are constantly being added to, that it is difficult to arrive at correct opinions in regard to their relative value for dental purposes. Prof. Miller, as you are aware, has done good work in this direction, and I quote eleven of these in the order of value :

| | |
|---|---|
| Mercuric Chloride. | Permanganate of Potassa. |
| Nitrate of Silver. | Eucalyptus Oil. |
| Iodoform. | Carbolic Acid. |
| Naphthaline. | Hydrochloric Acid. |
| Iodine. | Phenylic Acid. |
| Oil of Mustard. | |

The following are some that have been found valuable clinically: Resorcin, boric acid, naphthol, hydronaphthol, a preparation from B. naphthol, hydrogen dioxide (peroxide of hydrogen), thymol, quinolene, iodol, eugenol, eucalyptol, sanatas oil, salacylic acid, quinia, and heat. Many others, old and new, might be added, but I have preferred to give only those that have been thoroughly tested in pathological conditions of the mouth. Those named have quite different properties, and must be used with an intelligent conception of the object expected to be attained, as well as to their escharotic and possibly toxic qualities. The practical illustration of treatment in my hands is as follows: the canal is thoroughly injected first with peroxide of hydrogen as preliminary. This powerful oxidizer of organic matter prevents possible injury in cleaning out the debris of pulp tissue. The instruments used are passed through the alcohol flame to sterilize them. The canal is then thoroughly washed, either with mercuric chloride solution, 1 gr. to 4 oz. of water, or what I prefer, a naphthol solution, as follows:

℞ Hydronaphthol..............................gr. ij
Alcoholis...................................oz. ss
Aquæ destil.................................oz. iss. M.
Sol. filtered.

This is a very strong preparation, and should be used with caution to avoid irritation of the mucous membrane. It is not an escharotic, but exceedingly irritating. The next application is either iodine in crystals, as recommended by Dr. Litch, or, what I prefer, iodoform in the form of a paste.

℞ Iodoformi.................................gr. j
Zinci oxidi.................................gr. vj
Acidi carbolici, 25 per cent. sol..............q. s. M.

This retains its character, as previously stated, for an indefinite period, hydrogen sulphide having no effect upon it; differing in this respect from iodine, for the latter breaks up $H_2S$ into hydriodic acid, sulphur being deposited. Whether iodoform acts through its strong and persistent vapor or purely through its antiseptic properties is not understood; but probably through both, for, according to Bartholow: "The vapor of iodine, like chlorine, but in feebler degree, decomposes sulphuretted compounds." While I have made some efforts to determine the effect of iodoform chemically, its action in canals, in putrefactive conditions, still remains uncertain. Clinically the results are entirely satisfactory. The objection to this agent is its penetrating, lasting and very disagreeable odor. This may be removed in part by some of the essential oils; but I prefer to use it with the least possible admixture. Other substances have been introduced to take the place of this, as iodol and iodide of bismuth; but my experience does not warrant me in giving them equal place to iodoform. The paste is covered with a pledget of cotton saturated with a strong antiseptic of non-toxic character, as thymol or hydronaphthol in solution, and then followed with a temporary stopping of cotton and wax, sandarach and cotton, or gutta-percha. The latter, if used, should be left with a vent extending from the cotton to the surface of the filling to serve as an outlet for any excess of gas. When all odor of putrefaction has ceased, the canal is generally considered to be prepared for filling. When it is remembered that the entire dentine is permeated throughout with tubes, and each of these contains organic matter in a state of decomposition, and that the central canal is the common outlet for the drainage, it becomes a serious question whether it *is* ready for filling; certainly not without further treatment. As it is manifestly impossible to reach the contents of these tubuli except by imbibition of fluids, the selection of which must be based on certain qualities, it is clear that an antiseptic, however powerful, will not accomplish the object aimed at. It will necessarily be temporary in its results, and the discoloration of the body will be the final effect. The desired object can only be obtained by some agent or agents that will change the character of the decomposed tissue in the tubes and render it undecomposable. This will be found in the coagulators, and, of these, the chloride of zinc stands at the head of the list. This, from its affinity for moisture, will penetrate deeply and persistently. Putrescence cannot go on. Albumen thus treated and exposed to the atmosphere has remained unchanged for months. The canal is first closed at the apical foramen, at the shoulder, with either a small piece of cotton saturated with carbolic acid, or, if possible, with a small piece of gutta-percha. Cotton, saturated with chloride of zinc in solution, is then passed into the canal and allowed to remain there for several days. The object of the cotton or gutta-percha at the foramen is to prevent the action of the chloride on the pericementum. This action may be desired at times. Hence its use requires judgment. The further treatment must be governed by each individual case.

This article would be incomplete without noticing the plan of using compressed heated air, introduced by Dr. H. C. Register, of Philadelphia. In the treatment of canals it is unquestionably of great value and hastens the operation. Dr. Register states that it effectually destroys all germ life and that a tooth can be filled with entire safety at once after heated air has been forced into the tooth under pressure. The only question that I entertain in regard to it is, that if the heat be carried to a degree sufficient to destroy micro-organisms, it may possibly weaken the tooth by burning or drying out the organic matter of the dentine, and especially of the enamel. This result followed the use of metal for filling, flowing at a low heat as Wood's metal. The crowns were so much weakened that the walls frequently fell from the filling.

The filling of these canals is so well understood and so thoroughly exhausted of all novel features, that it might be omitted; but there are some points I wish to refer to briefly. Gold, tin and gutta-percha possess valuable qualities and have each in their way given excellent results. Argument seems to me to be wasted in attempts to discuss the relative merits of these materials. With suitable conditions each has its value and will preserve the tooth from the irritation of septic poison; but may not and, if my reasoning be correct, cannot prevent discoloration as neither of them can affect the organic tissue of the tubes. For the reason given under the consideration of chloride of zinc, I prefer the oxy-chloride of zinc for filling canals.

There has been adopted in recent years a mode of treatment that seems to me to require special notice; as, in my opinion, it is not only unscientific but reprehensible. I allude to the filling of canals with cotton saturated with carbolic acid. The advocates of this practice contend that success justifies the means adopted. This, if it were true, might be a satisfactory argument; but it has yet to be proven, while there are many facts in direct opposition to any such claim. Carbolic acid, as well as all germicides, has a limit to its power, the effect in time ceasing. It is true that both carbolic acid and creosote are coagulators; but they act very slowly in this direction and it is questionable whether either of them will change to any depth the contents of the tubes. With the loss of germicidal power there comes a time when the cotton will become offensive and a constant element of danger to the tooth. Cotton is an efficient filter and is, therefore, valuable to prevent the ingress of the micro-organisms from the atmosphere; but is not to be relied on for continued duty in a tooth canal. It is not a serious matter in the form of a small pellet, saturated with carbolic acid and placed at the constricted portion of the canal as it enters the apical foramen and may there have a certain value in protecting the pericementum; but beyond that I regard it as objectionable.

The importance of pulp treatment in connection with pericemental inflammation has not, that I am aware of, been noticed as its importance demands. The pathological state which we denominate pericementitis is the result of pulp disorganization by some of the well known forms of destructive lesions. To undertake the reduction of this inflammation, locally or combined with systemic treatment, must result in partial or complete failure if no attention be paid to the pulp. Inflammation of the pericementum at the apical foramen, of a pathological character, is certainly impossible unless the central and formative organ be first involved. The cause of the lesion of the investing membrane lies in the canal and whether it be acute pulpitis or from decomposed particles, the treatment must begin with the canal and this must be accorded the careful management heretofore described. The satisfactory results growing out of this treatment has robbed this once cause of anxiety of nearly all the difficulties and reduced the loss of teeth, in my hands, to a small fraction of former years.

The conclusions arrived at in this paper may be condensed as follows:

1st. That in the forceful removal of the pulp we have the only condition that favors immediate filling.

2d. That death by escharotics or by inflammation necessitates treatment.

3d. That antiseptic agents will not prevent discoloration of dentine and, therefore, an agent must be used to change the organic tissue in the tubes into an insoluble compound.

4th. That while gold, tin and gutta-percha are each good for filling canals, oxychloride of zinc is to be preferred for its valuable and persistent coagulating property.

5th. That pericementitis cannot be successfully overcome without first treating the primal cause, the diseased pulp, antiseptically.

